# ICD support groups: Are they still relevant now?

**DOI:** 10.1016/j.hroo.2025.03.021

**Published:** 2025-04-03

**Authors:** Samuel F. Sears, Samantha McCrary, Tiffany Andrade, Lauren Rousseau, Ann Kirkness, Allana Fantin, Julie B. Shea

**Affiliations:** 1 Department of Psychology, East Carolina University, Greenville, North Carolina; 2 Department of Cardiovascular Sciences, East Carolina University, Greenville, North Carolina; 3 Brigham and Women’s Hospital, Boston, Massachusetts; 4 Royal North Shore Hospital, Sydney, Australia; 5 Alberta Health Services, Calgary, Canada

**Keywords:** Implantable cardioverter-defibrillators, Support group, Cardiac-specific distress, Patient acceptance, Mental health

## Abstract

While implantable cardioverter-defibrillators (ICDs) save lives in patients at risk for potentially life-threatening arrhythmias, ICD shocks may prompt fear and anxiety and cause potential psychological trauma in patients. Since the beginning of the ICD’s broad use, healthcare professionals recognized the unique demands and experiences associated with this therapy. Some clinics responded by initiating patient-focused meetings broadly referred to as “ICD support groups” to address the mental health challenges in these patients. Decades of research have now underscored the importance of ICD patients’ psychological and behavioral processes and their associations with adverse health outcomes. ICD patients frequently report disease-specific mental health symptoms, including ICD shock anxiety, poor device acceptance, body image concerns, and decreased physical activity, that require unique clinical expertise from professionals to effectively manage. The original impetus for ICD support groups persists, but relatively few ICD support groups remain active. Online offerings may have taken on some of these functions. We suggest that ICD support groups remain a strategic intervention to address patient psychological and lifestyle concerns. This paper reviews the value of ICD support groups in the United States, Australia, and Canada and analyzes the current state and potential value of ICD support groups.

**Key Findings**

▪ Implantable cardioverter-defibrillator (ICD) support groups were an innovative response to patient needs from the early use of the ICD.
▪ A variety of barriers impede the development and maintenance of support groups, including failure to reimburse clinicians, start-up costs, lack of institutional support, patient attrition, and competing clinic demands.
▪ Because the need for support persists, other methods of support have arisen, such as online support groups on various platforms like Facebook and YouTube.
▪ While comprehensive psychosocial care may remain aspirational for many clinics, the ICD support group is one highly efficient tactic to effectively and efficiently address the numerous patient concerns that can arise.

## Background

The establishment and broad use of the implantable cardioverter-defibrillator (ICD) as a lifesaving primary and secondary prevention device for sudden cardiac arrest marks a significant medical achievement. The invention of this device as a therapeutic modality that could monitor, evaluate, and treat potentially life-threatening arrhythmias ushered in an exciting era of technology. Simultaneously, challenges arose in patients who were coping with the reality of spontaneous arrhythmias and device therapies (eg, ICD shocks) that could trigger fear and anxiety and potentially cause psychological trauma.^1^

Cardiac electrophysiology (EP) practices pride themselves on technical precision and clinical excellence, but accessing effective psychological care to address patient mental health concerns proved a novel and challenging problem to address. Empathic EP providers developed ICD support groups to address these emerging patient concerns.^2^ No national or international estimates are available about the waxing and waning of ICD support group availability from the past or present. This individual center clinical response was a reasonable effort to care for patients with ICDs but lacked any systematic best practice, form, or function. The only consensus was that patients with ICDs needed some degree of emotional and informational support to complement the technological advance of the ICD.

Extensive research over the past 30 years has established that patients benefit from the ICD, but they encounter specific challenges living with the ICD. Recent meta-analyses reviewing 109 studies with 39,954 patients indicated that 22.58% of patients with ICDs experience clinically significant anxiety, 15.8% depression, and 12.43% posttraumatic stress disorder.^3^ Moreover, a synthesis of qualitative studies indicated that fear and insecurity, the need for information, new impacts on life, living with ICD shocks, gender-specific concerns, and the role of family remain the major themes of concern for ICD patients.^4^ Interestingly, anxiety and depression have been linked to mortality but not to ICD shocks in a recent meta-analysis with 37 studies and 35,005 patients.^5^ Research has further substantiated ICD-specific patient reactions such as shock anxiety, poor device acceptance, body image concerns, and decreased physical activity that warrant clinical attention.^6^, ^7^, ^8^, ^9^, ^10^ ICD support groups appear to positively address some of these issues via sharing experiences and acceptance strategies focusing on common themes including confronting mortality, coping through sharing and learning, and providing space for discussion.^11^^,^^12^ Collectively, the substantial impact of the psychological processes and potential patient burden of living with cardiac disease, and an ICD remains critical and challenging to address.

The purpose of this paper is to review the value of ICD support groups and provide examples from recent support groups in the United States, Australia, and Canada.

## ICD support group examples

Enhancing the ICD patient experience has been a consistent aspiration for ICD clinics. In the following, we detail 3 different ICD support group examples from around the world. Each has unique aspects to their historical development and more recent efforts at sustainability. Table 1 details their history and characteristics.

**Table 1:** Details of ICD support group examples

	Clinic location		
	Boston	Calgary	Sydney
Established	1990s–2000; 2022–present	1989–present	1997–present
Distinction	Hybrid: virtual and in person	Province-wide reach with programming	Long-term operation in the cardiac rehabilitation area
Leadership	Physician assistants	Registered nurses	Registered nurses
Location	Independent practice	Cardiology service line	Cardiac rehabilitation

ICD = implantable cardioverter-defibrillator.

### Boston, Massachusetts

As medical professionals, we often prioritize the medical optimization of conditions that result in ICD placement, but we lack the resources to provide support for the mental well-being of patients living with chronic device therapy. Historically, the Brigham and Women’s Hospital (Boston, Massachusetts) arrhythmia team hosted ICD support groups for patients in the 1990s, but these meetings were discontinued in the 2000s due to a lack of resources and support. Thus, there was a recognized gap in mental health support for these patients, which was the impetus for this support group's re-creation.Table 2 presents a set of steps and lessons learned about establishing the support group in the current era.

**Table 2:** How to start an ICD support group at your center

Identify the goals of the group
• Educational • Emotional support • Social engagement • Connection of patients to professionals
Engage key leadership
• Provider leadership • Hospital and institutional leadership • Behavioral health leadership
Establish a planning committee to diversify education opportunities and moderate patient breakout sessions.
• This group may include patients, device manufacturer personnel, and healthcare team members (RNs, MDs, PAs, NPs).
Draft short- and long-term initiation and sustainability plan
• Considerations of meeting frequency, meeting location, meeting curricula, meeting speakers, meeting procedures, meeting publicity and recruitment, resources necessary for successful implementation
Seek institutional resources
• Practice, hospital, and foundational resources • Opportunity for benefactors to support • Establish a fund for any donations
Determine modality: in person, virtual, or hybrid
Review short- and long-term plan again with garnered resources
• Set the first meeting date approximately 6 months ahead • Lockdown agenda and speakers and run the meeting • Encourage strong staff and physician attendance • Consider catering refreshments • Consider practical limitations of ICD patients for parking and timing • Consider hospital, industry, and vendor exhibits
Publicize the meeting extensively
• Media, MyChart, clinic flyers, online groups
Ready, set, and support!
• Consider the first meeting a success for its occurrence • Provide opportunities for patient feedback
Debrief with key leadership
Plan for the support group 2

ICD = implantable cardioverter-defibrillator; NP = nurse practitioner; PA = physician assistant; RN = registered nurse.

We distributed a targeted patient survey via our electronic medical record to assess patient needs and hosted an inaugural ICD support group meeting in November 2022 as a hybrid event (in-person and virtual). Since November 2022, we have hosted 6 meetings, with a goal of 3 or 4 meetings/year. The meeting structure includes a guest speaker followed by breakout sessions to facilitate discussions and allow for patient interaction. The guest speakers have included patients, family members of patients, healthcare personnel, and device manufacturer team members who are patients themselves. Although we encourage in-person attendance to engage with other patients and staff, we find that about two-thirds of participants prefer to attend virtually. This is a great option, given the urban location of these events, and offers a greater geographical outreach. Each event concludes with an event evaluation to shape future sessions with patient-requested and supported initiatives.

To allow for increased diversity and expertise, we created a planning committee composed of patients, device manufacturer personnel, and healthcare team members (registered nurses, MDs, physician assistants, and nurse practitioners) to brainstorm breakout session topics and to facilitate patient-led discussions. Initially, committee meetings were established prior to each support group meeting; however, with increased events, meetings are only held as needed. Our continued goal is to provide a platform for patients to share stories, discuss adversities, and feel welcomed and supported in a cardiac device community.

Affiliation with cardiac psychiatry in this program has provided early referral for ICD patients with mental health care. Frequent, multimodal mental health treatment consisting of both pharmacological and psychotherapeutic interventions has been offered to these patients to treat device-related psychosocial distress and function while offering ongoing support and acute mental health care. Our ICD support group has allowed for improved understanding and awareness of our patients’ vulnerability, outlook, and psychological impact. This has provided an opportunity to foster the patient-provider relationship and improve our practice, both as a department and an institution.

### Sydney, Australia

The ICD support group forms an integral part of the services we offer under the cardiac rehabilitation (CR) “umbrella” at Royal North Shore Hospital (Sydney, Australia). While CR has a vital role to play in a patient’s recovery, providing support, education, supervised exercise, and ongoing monitoring of their physical and emotional well-being, it is clear that ICD patients can face some unique challenges and have more specific needs in terms of education and psychosocial support.

The ICD support group at Royal North Shore Hospital was established in 1997; our aim is to provide education and support specific to their needs and provide an opportunity for them to connect with other ICD recipients. Ongoing telephone and peer support is also available. ICD group education sessions are facilitated four times a year and cover a variety of topics relevant to ICD patients and their families. In 2013 a “subgroup,” the “Young ICD Network,” was also created (including a Facebook site), to provide information specific to the particular needs and interests of younger ICD recipients—for example, having a family, relationships, sexual intimacy, exercise, and work. In Australia, public hospitals are mainly funded by the Australian government, which means CR services (including the ICD support group) are fully funded and accessible to all Australians free of charge.

Previously sessions were run face to face, but COVID ushered in the digital era with the introduction of telehealth and online education. ICD-specific support groups are not widely available in Australia; thus, a “hybrid” model offering both online and face-to-face options enabled us to expand our reach and offer support options for ICD patients throughout rural, regional, and remote areas of Australia.

Integrating CR and ICD support groups programs has many advantages. It provides a multidisciplinary approach, offering an extended period of support and surveillance in conjunction with ICD-specific education. CR provides a natural point of entry for mental and psychological aspects of recovery that has been quite acceptable to patients in Australia.

### Calgary, Alberta, Canada

The ICD support group in Calgary, Alberta, Canada, began over 35 years ago with modest roots. Its inaugural meeting in 1989 was held in a coffee shop, led by 2 ICD patients affectionately calling themselves “The Lucky Tickers.” Over the decades, the group has navigated challenges and celebrated successes, driven by the shared commitment of cardiac device staff and patients to continuously improve and expand its reach.

Today, the ICD support group meets quarterly, facilitated by the dedicated volunteer efforts of cardiac device nurses and donated time of guest speakers from a variety of healthcare disciplines. Historically, leadership roles have also been held by patients, though key patient leaders have recently stepped down from these positions.

One of the group’s notable achievements is its emphasis on family-centered care, recognizing that successful living with an ICD is a collaborative effort. Meetings often include friends and family, with some sessions seeing nearly half of attendees being supporters rather than patients. Another success lies in the patient-driven structure of the meetings. Based on participant feedback, the group rotates meeting locations throughout the city to enhance accessibility and alternates between informal psychosocial support sessions and formal educational presentations featuring guest speakers.

Despite its successes, the group faces challenges, particularly in enrollment. Calgary is home to over 1,100 ICD patients, yet only approximately 2% actively participate in the group—a rate that research indicates falls short of meeting patient needs. To address this, the group is exploring strategies to broaden engagement, including hybrid meeting formats, transitioning from “opt-in” to “opt-out” email communications, and considering the creation of subgroups for specific demographics, such as younger ICD patients. The ICD support group remains dedicated to supporting patients and families, helping them live confidently and resiliently with their devices across the province of Alberta.

## Discussion

ICD support groups were an innovative response to patient needs from the early use of the ICD. The presented institutional examples demonstrate both restarted (Boston, Massachusetts) and long-term running (Sydney, Australia and Calgary, Canada) groups around the world continuing to address the ICD patient experience. In support groups, ICD patients can discuss challenges with their devices, collaboratively problem-solve issues, and learn about new educational resources, while receiving emotional support from others who share similar experiences. Interpersonal connectivity facilitates patients’ understanding of the universality and normality of their concerns. Table 3 provides examples of the ICD support group topics from our presented ICD support groups that highlight the breadth and depth of key issues covered in support group meetings.

**Table 3:** Suggested ICD support group topics for planning

Important topics to consider for support groups	
E/PA	• Safe E/PA practices • Importance of E/PA • Incorporating E/PA into your daily routine • Cardiac rehabilitation • Walking prescriptions
Pharmacology	• Common medications • Medication interactions • Importance of medical adherence • Discussing side effects • Provider Q&A
Psychology	• Optimism • Stress management/destress tolerance • Cognitive reframing • Successful living with your ICD • Confidence and resilience • Values-based living • Provider Q&A • Shock plan
Nursing	• Remote monitoring • Coordination of care • Device education • Medication reconciliation
Social support	• Discussing your device with your support network • How to ask for help • Use of online chat groups
Family support	• Managing family and device • Discussing your device with your family • Recreational activities
Community participation	• Community engagement resources (in person/online) • Cardiac disease awareness • Emergency medical discussions
Marriage/relationships	• Caregiver burnout • Spouse/significant other concerns • Role changes • Sex • Travel and recreation
Medical aspects	• Patient/provider relationships • Self-advocacy • Device education • Remote management • Role of ablation • Role of antiarrhythmic medications • Comanaging heart failure • Long-term considerations • End of life • Provider Q&A

E/PA = exercise/physical activity; ICD = implantable cardioverter-defibrillator.

Support groups across health problems and healthcare systems seem to be a desirable means to reach patients. Other patient disease groups, such as cancer, have broadly established support groups at most large clinical centers to meet patients’ educational and psychological needs more efficiently. A variety of barriers impede the development and maintenance of support groups, including failure to reimburse clinicians, start-up costs, lack of institutional support, patient attrition, and competing clinic demands. Without support groups, the opportunity for patients to learn about the unique aspects of their condition and device directly is somewhat limited, despite broad evidence of its utility. Table 4 displays some of the challenges and opportunities that a support group can provide.

**Table 4:** Considerations of the opportunities and limitations of ICD support groups

Opportunities	Limitations
1. Interpersonal connectivity	1. Participation/interest
2. Universalization and normalization of concerns	2. Start-up and ongoing costs
3. Acknowledgment from spouses and family members	3. Competing clinical interests
4. More efficient patient education	4. Provider reimbursement
5. Improved management of health-related fears	5. Facilitation time/interest
6. Anxiety relief from increased feelings of belongingness	
7. Improved device acceptance	
8. Reduced healthcare utilization	

ICD = implantable cardioverter-defibrillator.

Outcome studies of support groups are scant, have generally been underpowered to detect differences, lack control groups, and are subject to methodological weaknesses such as single-center studies, selection biases, and small sample sizes. Anxiety has been the most common target of clinical attention appropriately, and a hallmark feature of anxiety is the avoidance of triggers and content that may increase arousal. Recent meta-analyses suggested that support groups helped patients manage their fears related to their health status, provided some anxiety relief by increasing participants' feelings of belongingness, and improved patients' device acceptance.^6^ Recent surveys of 139 ICD patients indicated that 42% of respondents said that they would attend a support group if offered.^13^ Interestingly, patients reporting the most ICD concerns and shorter times since implantation were more likely to indicate that they would attend, suggesting a potential focus for support group programming to the patient’s early experiences with the ICD.

The unique challenges of the ICD patient appear to have been consistent over decades of research.^14^ Psychosocial care for ICD patients has consistently demonstrated efficacy for anxiety and depression.^15^ Additional research has indicated that the inclusion of family members may also improve the effects of psychosocial care.^16^ The availability of psychosocial care in cancer clinics is well documented. In fact, recent U.S. national survey results of 1145 cancer centers indicated that 85.4% offered mental health services.^17^ However, this type of commitment to mental health care for cardiac or ICD patients is suspected as comparatively very rare. Today, social media using websites like Reddit, Facebook, and chat rooms allow ICD patients many options to address their concerns. The convenience of the internet for some patients creates broad accessibility to ICD support forums run by patients. The future clearly points to online support via patient advocacy groups such as sudden arrythmia death syndrome, hypertrophic cardiomyopathy, and other patient-led sites like HeartWarriorProject.com. The ability for ICD patients to advocate and assemble experts from many disciplines for online content has been established. This virtual model roughly approximates the single-center ICD support groups of the past and present in this paper. Patient-led and professional-led groups are likely to continue because of the need. Evidence for professional-led groups remains promising. One study randomized ICD patients to an interactive virtual nurse-led ICD support group vs a control group to impact fatigue, shock anxiety, and device acceptance.^18^Follow-up evaluation at 1 month indicated significant differences in all 3 key variables. These recent data suggest that the flexible, interactive, and nurse-led approach can also impact the patient experience successfully. Patient-led groups likely will not conduct outcome studies, but this is the new age of communication. Patients have and need a voice to each other and professionals.

Modern EP practice continues to evolve, including advances in remote patient monitoring, advances in device technology, and innovative device programming limiting ICD shock exposure. ICD patients benefit broadly from remote monitoring for convenience and improved clinical outcomes.^19^ However, remote monitoring also potentially reduces the EP clinic’s face-to-face clinical interactions to discuss psychosocial concerns and frequently asked questions. ICD shock reduction is also a welcomed advance, but fears of ICD shocks and psychological recovery from ICD shocks persist.^20^ Clinicians must work to normalize conversations about mental health in EP clinics and develop strategies for screening and referral of psychological issues. Support groups represent an in-house strategy, but increased organizational commitment and plans for sustainability are needed. Combined efforts with patient advocacy groups to provide support and educational programming may provide a way forward as well.

## Conclusion

Due to ongoing patient interest, ICD support groups remain relevant today. The milieu of the EP clinic remains dedicated to high levels of technical expertise, device-based programming, medical management, and the ongoing need for patients and families to feel safe and confident about their ICD providing protection from potentially life-threatening arrhythmias. While comprehensive psychosocial care may remain aspirational for many clinics, the ICD support group is one highly efficient tactic to effectively and efficiently address the numerous patient concerns that can arise. ICD patients experiencing disease-specific distress deserve tailored care to meet their unique mental health needs. Support groups combine social, psychological, and medical assistance to target this population’s complex disease profile. Though support groups are not the only option for mental health intervention in this population, they are often a more accessible resource for patients because many are no-cost or community-run services. Online support groups may also be an attractive option for individuals who may not have access to reliable transportation or who are hesitant to engage in traditional mental health care. Recognition and support of the ICD support group by institutions, industry, and even health policy could increase the chances of ICD support groups flourishing and fully addressing patient needs.

As noted previously, Tables 2 and 3 provide an outline for starting a support group and suggest several topics for discussion. To promote patient satisfaction, clinics should consider making additional accommodations to ensure patient privacy and collect members’ feedback. Both in-person and online support groups typically establish group norms before every meeting. The first norm of support group is always confidentiality. Members are reminded that the content discussed in group is to remain confidential but should acknowledge that only providers are bound to confidentiality rules. In online settings, clinics might consider establishing additional privacy protections, including requiring patients to attend the session in a private room with their video cameras on to hold members accountable for protecting the privacy of the group. There is likely no need for a formal evaluation of support groups. Metrics like routine attendance, participant engagement, and positive impressions will illuminate members’ satisfaction with the group. Any step that practitioners take to demonstrate their dedication to their patients beyond time-pressed clinical visits will likely leave them with a positive impression. Support groups are a highly efficient way for clinics to promote the emotional well-being of their patients.
